# Description of the Vasospasm Phenomena following Perimesencephalic Nonaneurysmal Subarachnoid Hemorrhage

**DOI:** 10.1155/2013/371063

**Published:** 2013-12-28

**Authors:** Daphna Prat, Oded Goren, Bela Bruk, Mati Bakon, Moshe Hadani, Sagi Harnof

**Affiliations:** ^1^Sackler Faculty of Medicine, Tel Aviv University, Ramat Aviv 69978, Israel; ^2^Department of Neurosurgery, Sheba Medical Center, 52621 Tel-Hashomer, Israel; ^3^Department of Radiology, Sheba Medical Center, 52621 Tel-Hashomer, Israel

## Abstract

*Background*. Perimesencephalic nonaneurysmal subarachnoid hemorrhage (PM-NASAH) is characterized by a benign course compared with aneurysmal SAH. While vasospasm (VS) after aneurysmal SAH is considered responsible for serious complications, VS post-PM-NASAH is not well documented. Our purpose was to characterize the incidence and course of VS among 63 patients—one of the largest databases of PM-NASAH patients with documented blood flow velocities in the literature. *Methods*. Data from 63 patients that were admitted with PM-NASAH from 2000 to 2012 and underwent transcranial Doppler tests to assess cranial vessel flow velocity was analyzed. *Results*. On average, the maximal flow velocity was measured on the 7th day after hemorrhage. Higher risk for VS was associated with younger age, female sex, and higher Hunt and Hess scores, a lower risk for patients treated with statins (*P* < 0.05). Using velocity thresholds for diagnosis of VS, 49.2% showed evidence of VS. This is the first description of blood flow velocities in PM-NASAH. VS average onset was on the 4th day, average cessation on day 15 after hemorrhage. No patients showed clinical manifestation of VS. *Conclusions*. VS post-PM-NASAH is not as rare as previously believed. However, its lack of clinical significance raises questions regarding the need for diagnosis and may suggest a less intensive treatment protocol.

## 1. Introduction

Nontraumatic subarachnoid hemorrhage (SAH), comprising twenty percent of all SAH [1], has an estimated annual incidence of 6 cases per 100,000 persons in the United States [2]. Spontaneous SAH is most often associated with ruptured intracranial aneurysms (70–80%) and less frequently with arteriovenous malformations (4%) [1]. However, in approximately 15% of all patients presenting with spontaneous SAH, the cause of the bleeding remains unknown even after detailed imaging studies [3, 4].

In two-thirds of SAH cases with normal imaging studies, the blood accumulates predominantly around the midbrain [5]. Such bleeding patterns were first described by van Gijn and colleagues in 1985 [6] and were termed perimesencephalic nonaneurysmal subarachnoid hemorrhage (PM-NASAH). The distinct radiographic pattern of PM-NASAH comprises of a hemorrhage located anterior to the midbrain or the pons, with or without an extension of blood around the brainstem, the suprasellar cistern, or the proximal sylvian fissures [2].

The etiology of PM-NASAH has not yet been determined [5]. Given its typically focal site and low volume of bleeding, it is likely that PM-NASAH represents the rupture of low-flow entities, such as a local venous or capillary structure, a perforating artery, a low-flow vascular malformation, or a capillary telangiectasia [7]. Compared with aneurysmal SAH (ASAH), PM-NASAH is associated with a better outcome, but recurrent bleeding and delayed cerebral ischemia may occur [8].

Although VS following ASAH is well documented, VS in the context of PM-NASAH is believed to be rare, with few reports present in the literature [7]. The pattern of VS in PM-NASAH is typically confined to the posterior circulation, although diffuse VS has been described [9]. Within a small subgroup of PM-NASAH patients who develop angiographic evidence of VS, only a small number of individuals become clinically symptomatic [7]. Rinkel and colleagues reported on a series of 65 patients with PM-NASAH, in which only 3% had angiographic evidence of VS [10]. In a smaller series of 20 patients, van Calenbergh and colleagues noted that although 20% of patients had angiographic VS, only one patient has developed neurologic deficit [11].

While PM-NASAH is considered as a subtype of angiographically negative SAH (AN-SAH), it is important to distinguish the clinical significance of VS in these groups. This difference is illustrated by Gross et al., in which among 77 patients who were evaluated with delayed angiography, 26% suffered from VS, 4% developed delayed infarcts, and 4% deteriorated due to delayed cerebral ischemia [12]. A diffuse hemorrhage pattern and a higher Hunt and Hess (H&H) grade were found to be risk factors for these complications. Hui et al. reviewed 94 patients with AN-SAH and found that the subgroup of patients with PM-NASAH was associated with a better outcome compared with the rest of the subgroups, with decreased risk for vasospasm and hydrocephalus [13]. Andaluz and Zuccarello found that compared with PM-NASAH, other types of AN-SAH were associated with significantly longer hospitalization and intensive care unit stay, greater complication rates, and worse outcomes [14].

The aims of the current study were to accurately assess the flow velocities in PM-NASAH and to describe, for the first time, the phenomenon of VS following PM-NASAH in the largest cohort of PM-NASAH patients with documented blood velocities established up to date.

Another aim was also to define a workup algorithm for VS, which provides the early diagnosis of clinically significant VS, and by thus decrease morbidity without prolongation of hospital stay.

## 2. Methods

### 2.1. Study Design

We used a retrospective cohort design in order to assess the risk factors for VS and its clinical outcomes after PM-NASAH.

### 2.2. Setting

We retrieved data from a prospective database that consisted of demographic data, clinical data, and transcranial Doppler (TCD) parameters for patients with SAH, admitted to the department of Neurosurgery at Sheba Medical Center, Tel-Hashomer, Israel, from January 1, 2000, to October 31, 2012. Data was compiled in accordance with the local IRB—Helsinki committee number 8199-10.

### 2.3. Participants

63 patients met the following inclusion criteria: (1) a computed tomography (CT) scan performed on admission showed a perimesencephalic (PM) pattern of hemorrhage, (2) without an aneurysm as a cause of the bleeding, on angiographic analysis. A PM pattern of hemorrhage was defined as (1) an evidence of bleeding in the PM, interpeduncular and prepontine cisterns, (2) without involvement of the brain parenchyma, the ventricular system, or the sylvian fissure, and (3) without indication of an aneurysm or other source of bleeding. A mild extension of the bleeding into the proximal sylvian fissure was still considered as a PM bleeding and was named PM Plus (PM+). Pregnant women, patients under the age of 18 or over the age of 80, and patients lacking judgment were excluded from this study.

### 2.4. Variables

For each patient, we recorded demographic data, smoking habits, and concurrent diseases such as hypertension or diabetes. We collected the following data for each hemorrhage: date of onset, symptoms and signs at the time of admission, possible triggers, and clinical grading at the time of admission according to the H&H grading scale [15]. We also recorded the imaging modalities performed for each patient, the treatment received, and the clinical outcome.

Informed consent was waived by the local IRB for a retrospective study.

### 2.5. Data Sources and Measurement

Data regarding demographics, smoking habits, and concurrent diseases was delivered by the patients. Data regarding hemorrhage details (onset, symptoms and signs, and H&H score) was recorded by the patients' physicians.

#### 2.5.1. Measuring Vasospasm

TCD is based on the principle that, given a constant cerebral blood flow, the flow velocity is inversely proportional to the vessel lumen cross section area. TCD is noninvasive, it is repeatable and inexpensive, and it can be performed at the patient's bedside. The most recent 2012 American Heart Association guidelines for the management of ASAH state that TCD is a reasonable method to monitor the development of arterial VS [16].

In the current study, a single trained operator (Bela Brule) performed all TCD examinations for all patients. TCD was performed daily as a part of routine follow-up examinations of all patients with spontaneous SAH (aneurysmal and nonaneurysmal).

Angiography (DSA or CTA) was performed on admission to rule out aneurysm or other vascular malformation that could be the cause of SAH and was repeated mostly in cases of PM+ type of SAH.

Flow velocities were measured in the distal portion of the extracranial internal carotid artery (ICA), proximal and distal middle cerebral artery (MCA), anterior cerebral artery (ACA), posterior cerebral artery (PCA) and vertebral artery (VA) bilaterally, and proximal and distal basilar artery (BA).

We used specific threshold criteria to define arterial VS, based on previously published reports [17–22]. The criteria included a mean flow velocity (MFV) in the intracranial ICA, MCA, and ACA greater than 120 cm/sec [18, 19]; a MFV in the VA greater than 80 cm/sec [20]; a MFV in the BA greater than 85 cm/sec [20]; a MFV in the PCA greater than 110 cm/sec [20, 21]; an extra-/intracranial ratio 2 for the carotid watershed (MCA, ACA, or intracranial ICA) greater than 3 [18]; and a proximal/distal ratio for the vertebrobasilar (VB) system greater than 2 [22].

TCD was performed daily to document velocities and to detect VS. VS and its course was observed and documented based on the following criteria. The day of VS onset and remission, the day of maximal velocity, the measured velocities, and the clinical outcome and treatment. Treatment was administered according to the treating physician (either Sagi Harnof or Moshe Hadani) and adhered to the protocol for active treatment of clinical VS only. Close monitoring was performed for TCD based VS or documented increased velocities.

### 2.6. Bias

Due to its retrospective design the study is inherently influenced by selection bias and information bias. No other bias as far as we are aware have influenced the results.

### 2.7. Study Size

The number of cases in the area during the study period determined the sample size.

### 2.8. Quantitative Variables

As mentioned above, the clinical significance of VS in PM-NASAH has yet to be determined. As such, we have chosen to describe VS using the accepted thresholds as well as by measuring the flow velocities of the intracranial arteries for all patients and identifying correlations between the velocities and patient characteristics.

### 2.9. Statistical Methods

Quantitative and continuous variables were described using sample size, mean, median, minimal and maximal values, and dispersion variables. Categorical and discrete variables were described using group size and observed and relative frequencies. Statistical analysis was performed with SPSS 20 for Windows (SPSS, Chicago, IL). The Chi-square test, Pearson's correlation, and Student's *t*-test were used. A *P* value <0.05 was considered significant.

## 3. Results

### 3.1. Participants

Among all patients admitted to our department with PM-NASAH during the study period, 63 patients met the inclusion criteria and were included in this study.

### 3.2. Descriptive Data

Among all 63 patients, 29 (46%) were females. The youngest patient was 24 years old and the oldest 75. The average age of our patients was 52.4. Hunt and Hess score on admission was 1 for 48 (76.2%) patients and higher for the rest (Table 1). Thirty-three (52.4%) of our patients were not reported to have vascular risk factors such as hypertension or diabetes mellitus. A total of 241 TCDs were performed for all patients, with an average of 3.76 TCDs performed per patient.

### 3.3. Outcome Data

As mentioned previously, we used velocity thresholds to diagnose possible VS. Using these thresholds, 31 of the 63 patients (49.2%) exhibited VS. Among these patients, more than three-quarters (24 cases—77.4%) had VS documented in the distal BA; other arteries in which VS was observed were the MCA (38.7%), proximal BA (16.1%), ACA (12.9%), PCA (9.67%), and VA (6.4%) (Table 2).

### 3.4. Main Results

#### 3.4.1. Vasospasm Defined by Velocity Thresholds

The onset of VS occurred between the 2nd and the 8th day after hemorrhage, with the average being on the 4th day. The spasm ended between the 9th and 25th day after hemorrhage, with the average being the 15th day. Although the maximal velocity for all patients was measured between the 2nd and 16th, with the average being the 7th day after hemorrhage, in the VS patient group, the maximal velocity was measured between the 5th and the 16th day after hemorrhage, with the average being the 8th day after hemorrhage.

We examined the differences between patients in the VS and non-VS groups. Using Student's *t*-test analysis to examine the relationship between age and vasospasm, we found that younger patients were more susceptible to VS (6.84 years younger on average, *P* = 0.008). No difference was found for sex, H&H score, vascular risk factors, smoking, and the season of the year in which the hemorrhagic event took place.

### 3.5. Other Analyses

#### 3.5.1. Flow Velocities in PM-NASAH

We examined the correlations between the mean flow velocities, patients, and SAH characteristics. Using Pearson's correlation, we found a negative correlation between the mean flow velocity in the distal BA and distal BA/VA and patient age (*P* = 0.03 and 0.02, resp.). This finding correlates with the results mentioned above, showing a possible higher risk for VS in younger patients. When comparing flow velocities and patient sex using Student's *t*-test analysis, we found that the mean flow velocities among women were higher, when measured on the ICA, VA, and proximal BA (*P* = 0.001, 0.014, and 0.001, resp.). Correlating the flow velocities with the H&H score using Student's *t*-test analysis, we found a higher mean flow velocity in the VA artery in the patients with H&H score of 2, 3, and 4 at admission, compared with patients with H&H score of 1 (*P* = 0.027).

We examined the relationship between statins treatment during hospitalization and VS and found a lower mean flow velocity in the ICA, ACA, VA, proximal BA, and distal BA arteries in patients treated with statins versus patients untreated with statins (*P* = 0.002, 0.035, 0.007, 0.008, and 0.013, resp.). No significant correlations were found between flow velocities and the following variables: smoking, season of the year, treatment with nimodipine during hospitalization, or other vascular risk factors. Overall, we found a significantly higher probability for VS with younger age, female sex, and a higher H&H score at the time of admission. Our findings suggest significantly lower risk for patients treated with statins. The minimum, maximum, and average flow velocities measurements in the different intracranial arteries are summarized in Table 3.

**Figure 1:**
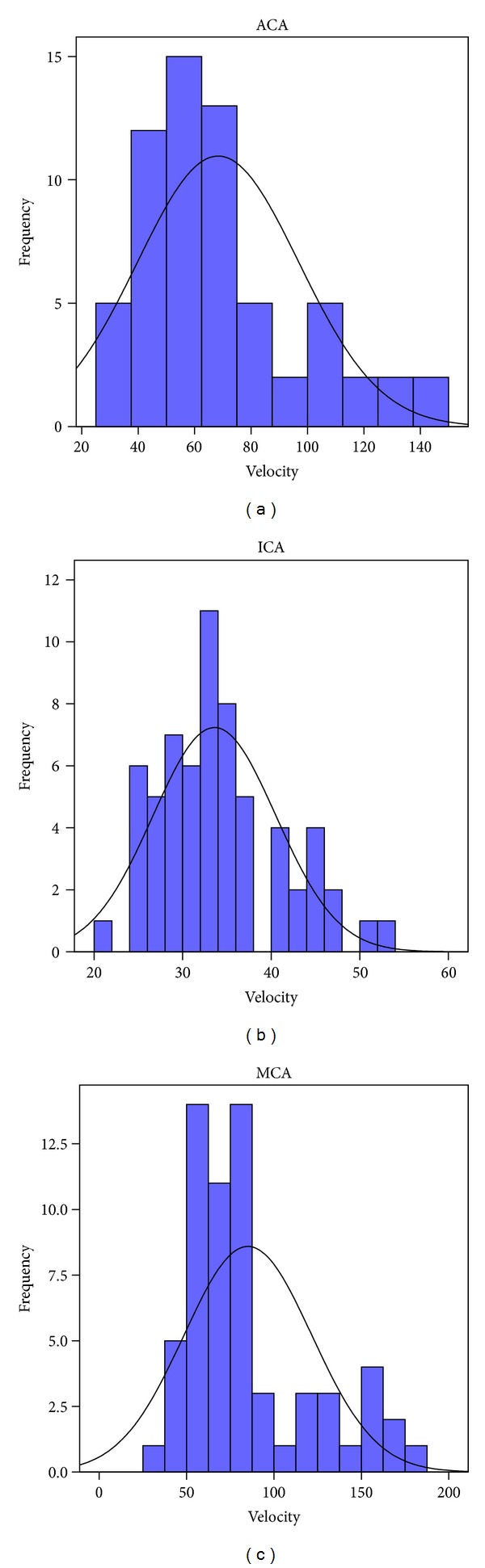
Flow velocity distributions in the anterior circulation (cm/sec). (MCA: middle cerebral artery; ICA: internal carotid artery; ACA: anterior cerebral artery.)

**Figure 2:**
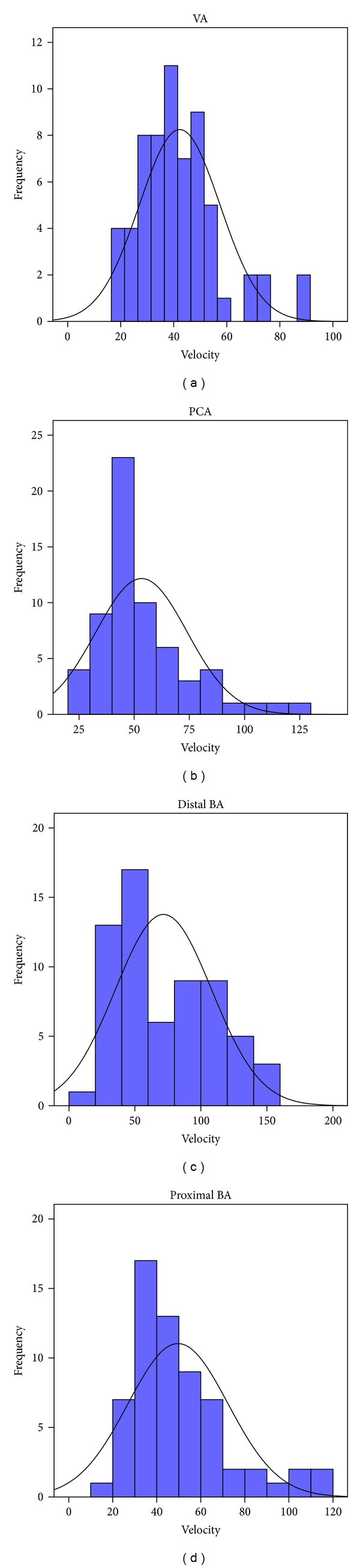
Flow velocity distributions in the posterior circulation (cm/sec). (PCA: posterior cerebral artery; VA: vertebral artery; BA: basilar artery.)

**Figure 3:**
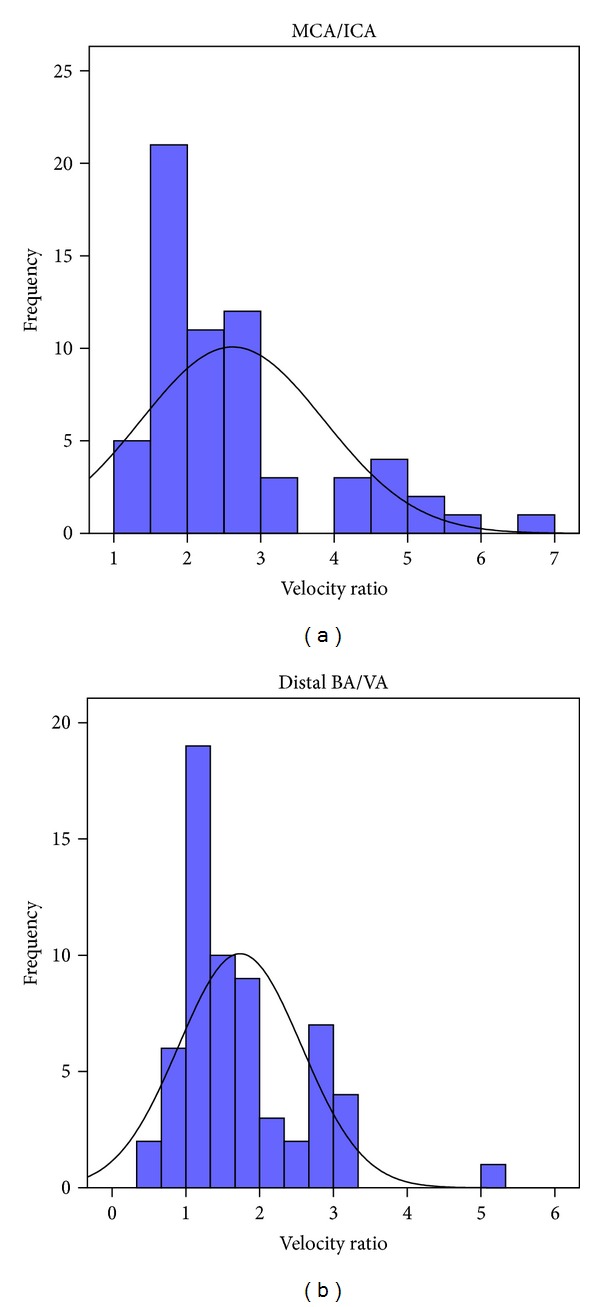
Flow velocity ratios. (MCA: middle cerebral artery; ICA: internal carotid artery; VA: vertebral artery; BA: basilar artery.)

Figures 1, 2, and 3 summarize the flow velocity distributions. The day of maximal velocity was between the 2nd and the 16th day after the initiation of the hemorrhage, with the average being the 7th day.

#### 3.5.2. Clinical Impact of Vasospasm

Symptomatic patients were categorized into two groups. The first group of patients exhibited symptoms that were recorded upon initial examination and were usually related to cranial nerves (CN) involvement (6 patients had CN involvement; CNs 3, 6, 7, or 8 were involved). The second group of patients had presented with a worsening headache that developed during hospitalization and responded to medical treatment including drainage of a cerebrospinal fluid (CSF). Nevertheless, none of the patients demonstrated symptoms that could be attributed to arterial VS, and none had manifestation of VS that could be tracked clinically.

## 4. Discussion

### 4.1. Key Results

The current study characterizes, for the first time, the phenomena of VS following PM-NASAH, in one of the largest cohorts of patients with PM-NASAH described in the literature. We described the VS phenomenon in PM-NASAH as well as the arterial flow velocities among our patients. We examined correlations between these variables, patient characteristics, and SAH characteristics. We found possible risk factors and showed the possible benefit from treatment with statins.

### 4.2. Limitations

This study presents a historical cohort group of patients diagnosed and treated in retrospect, without the guiding hand in the data collection during the hospital stay. In addition, the study lacks long-term followup for patients, referring to the clinical implications of the described objective findings.

### 4.3. Interpretation

Our series shows that VS in PM-NASAH is different from VS after ASAH in several aspects (Table 4). Most notably, VS after PM-NASAH is less common than after ASAH, and its clinical significance is negligible in comparison to ASAH VS. In our series, 24 of all 63 patients (38%) had VS detected by TCD examination in the distal BA; however, the locations of the VS or the number of arteries involved had no clinical impact. The onset day, maximal spasm day, and duration are similar to those in ASAH. These details emphasize the importance of establishing a protocol for monitoring and treating PM-NASAH.

### 4.4. Generalisability

The lack of clinical significance of VS in the PM-NASAH patients in our series resembles to the results of other studies. As such, we suggest that using a velocity threshold for VS in PM-NASAH has little significance. We strongly recommend clinical observation and clinically-based decision making regarding VS in the setting of PM-NASAH. The extremely low incidence of neurological deficit subsequent to VS should be taken under consideration when handling and observing PM-NASAH patients. Early discharge of these patients, based on the belief that ischemic symptoms will not occur, may be inadequate, since the exact incidence of PM-NASAH VS is still unknown and requires further investigation. We concur with the results of other studies [24] that early discharge of these patients may be suitable if adequate postdischarge observation is available from carefully trained observers. The proposed follow-up protocol for the patients with PM-NASAH includes baseline complete TCD examination within 24 hours after patient's admission and repeated TCD examinations only in cases of clinical symptoms, suggestive of vasospasm-related complications.

Angiography (DSA or CTA) should be performed on admission to rule out aneurysmal origin of SAH. Repeated angiographic examinations may be needed primarily in cases of PM+ for the same purpose.

## 5. Conclusion

To date, PM-NASAH VS is rarely cited in the literature. This complication is not as rare as previously thought, with almost 50% of patients with PM-NASAH examined in this series having imaging evidence of VS. However, the lack of its clinical significance raises questions regarding the need for diagnosis using the flow velocity threshold, as used in ASAH. The benign course of VS in the context of PM-NASAH may suggest the use of a less intensive treatment protocol in comparison to ASAH. Early discharge may be considered, if adequate observation out-patient clinic is available. Nevertheless, although not exhibited by our patients, rare but dangerous sequelae are possible, and further research is needed in order to gain a more comprehensive understanding of the risks of the apparent benign nature of this condition.

## Figures and Tables

**Table 1:** Clinical features of study group.

Variable	Patients
Total patients number	**63 (100%)**
Sex	
Male	34 (54%)
Female	29 (46%)
Hunt and Hess score on admission	
I	48 (76.2%)
II	12 (19%)
III	2 (3.2%)
IV	1 (1.6%)
Age	
Minimal	24
Maximal	75
Average	52.44
Vascular risk factors	
None	33 (52.4%)
Hypertension	19 (30.2%)
Diabetes Mellitus	9 (14.3%)
Other	2 (3.2%)
TCD performed	
Total	**241**
Per patient	
Minimal number of exams	2
Maximal number of exams	8
Average number of exams	3.76

**Table 2:** VS defined by velocity thresholds—distribution by arteries involved.

Variable	Patients
Artery in which VS was detected	
Distal BA	24 (77.4%)
MCA	12 (38.7%)
Proximal BA	5 (16.1%)
ACA	4 (12.9%)
PCA	3 (9.6%)
VA	2 (6.4%)
ICA	0
Number of arteries involved per patient	
1	19 (61.4%)
2	5 (16.1%)
3	5 (16.1%)
4	1 (3.2%)
5	1 (3.2%)
Total	**31 (100%)**
Average	1.71

ICA: internal carotid artery; MCA: middle cerebral artery; ACA: anterior cerebral artery; PCA: posterior cerebral artery; VA: vertebral artery; BA: basilar artery; VS: vasospasm.

**Table 3:** Summary of the minimum, maximum, and average flow velocities in the intracranial arteries.

Artery	Minimum (cm/sec)	Maximum (cm/sec)	Mean (cm/sec)	Std. deviation (cm/sec)
ICA	21	52	33.59	6.95
MCA	34	184	85.08	36.56
MCA/ICA	1.06	6.75	2.62	1.25
ACA	25	146	68.32	28.66
PCA	24	127	53.33	20.66
VA	19	90	42.25	15.23
Proximal BA	18	116	49.63	22.78
Distal BA	18	153	71.67	36.50
Distal BA/VA	0.59	5.05	1.73	0.83

ICA: internal carotid artery; MCA: middle cerebral artery; ACA: anterior cerebral artery; PCA: posterior cerebral artery; VA: vertebral artery; BA: basilar artery.

**Table 4:** Vasospasm after PM-NASAH versus ASAH.

	PM-NASAH VS in our series	ASAH VS in the literature
Incidence	49.2%	70% [23]
Clinical significance	None	Ischemic neurological symptoms in 50% of large artery VS [16]
Spasm onset*	4.9 d	3 d [23]
Day of max. velocity*	7.03 d	6–8 d [23]
Spasm resolution*	15.5 d	2-3 weeks [23]

*Average number of days/weeks after hemorrhage.
